# Dry eye in rheumatoid arthritis patients under TNF-inhibitors: conjunctival goblet cell as an early ocular biomarker

**DOI:** 10.1038/s41598-020-70944-9

**Published:** 2020-08-20

**Authors:** Fany Solange Usuba, Ana Cristina de Medeiros-Ribeiro, Priscila Novaes, Nadia Emi Aikawa, Karina Bonfiglioli, Ruth Miyuki Santo, Eloisa Bonfá, Milton Ruiz Alves

**Affiliations:** 1 Department of Ophthalmology, Hospital das Clinicas HCFMUSP, Faculdade de Medicina, Universidade de Sao Paulo, Sao Paulo, Brazil; 2 Rheumatology Division, Hospital das Clinicas, HCFMUSP, Faculdade de Medicina, Universidade de Sao Paulo, Sao Paulo, Brazil

**Keywords:** Cell biology, Biomarkers, Diseases, Health care, Medical research, Rheumatology, Signs and symptoms

## Abstract

Dry eye disease (DED) is common in Rheumatoid Arthritis (RA) patients. The application of conjunctival goblet cell count as a clinical biomarker to diagnose and respond to treatment can take place in rheumatoid arthritis patients under TNF-inhibitors (TNFi) therapy. This study aimed to investigate the ocular surface parameters and the long-term effects of TNFi therapy on ocular surface features and goblet cell count of rheumatoid arthritis patients. At baseline, rheumatoid arthritis patients eligible to TNFi were compared to healthy controls (similar age/gender), regarding Ocular Surface Disease Index (OSDI) questionnaire, Schirmer I test, tear break-up time test, vital dye staining of the ocular surface, and conjunctival impression cytology. DED severity grade, impression cytology score, and goblet cell count were analyzed. Rheumatoid arthritis patients were followed after three (3 M) and 12 months (12 M), during TNFi treatment. Sixteen rheumatoid arthritis patients and 24 controls were compared: a higher frequency of abnormal OSDI (68.8% vs. 16.7%, *p* = 0.002), Schirmer’s test < 10 mm (37.5% vs. 8.3%, *p* = 0.042), meibomian gland dysfunction (50% vs. 8.3%, *p* = 0.007), abnormal impression cytology (75% vs. 8.3%, *p* < 0.001), and mild to moderate DED (81.3% vs. 4.2%, *p* < 0.001) were observed in rheumatoid arthritis patients, who also had lower goblet cell count [325 (274–707) cells/mm^2^ vs. 742 (562–863) cells/mm^2^, *p* = 0.004]. The presence of Meibomian gland dysfunction was associated with higher disease activity scores (*p* < 0.05). The prospective early observation of these patients at 3 M showed an increase improvement in tear production by Schirmer’s test [13 (7.5–17.5) vs. 23.5 (16–35); *p* = 0.001], and an improvement in impression cytology score [1 (0.5–2) vs. 1 (0–1), *p* = 0.031] and in goblet cell count [325 (274–707) vs. 931 (656–1,244), *p* < 0.001]. Eight RA responders to TNFi were also re-evaluated at 12 M with further improvement in goblet cell count [393 (275–827) vs. 872 (502–1,185) vs. 1,079 (867–1,244), *p* = 0.047]. Multifactorial DED is frequent in RA patients, comprising aqueous, lipid, and mucin components. TNFi prompt improves tear production and recovers the goblet cells, which can be a biomarker of the pathological process and response to therapy in this population.

## Introduction

Rheumatoid arthritis (RA) is the most common cause of chronic inflammatory arthritis of unknown etiology that primarily targets synovial tissues but includes extra-articular manifestations^1,2^. Ocular manifestations, particularly keratoconjunctivitis sicca (KCS), may occur independently of RA disease activity and should be evaluated in all patients with RA regardless of other articular or extra-articular disease expressions^3^.

Keratoconjunctivitis sicca (KCS) or dry eye disease (DED) is a common cause of ocular morbidity worldwide, affecting 5% to 50% of the population and about 15–90% in RA patients^3–5^. Dry eye disease presents low volume and quality of the tear film and can hinder daily activities, harm visual function, damage the ocular surface, and consequently, the quality of life of patients^6,7^.

DED’s pathogenesis is not entirely understood, but the loss of homeostasis of the tear film (tear film instability and hyperosmolarity), ocular surface inflammation and subsequent damage, and neurosensory abnormalities have a pivotal role in the disease^8^. Chronic inflammation leads to conjunctival pathologic changes such as squamous metaplasia and goblet cell loss demonstrated by cytological analysis of the conjunctiva from patients with DED^9^.

The successful joint outcomes associated with TNF-inhibitors (TNFi) therapy may provide a potential new modality for DED treatment. Murine models suggested that TNF blockers effectively suppressed the lacrimal gland and corneal inflammation^10^. However, only a small case series demonstrates that TNF-inhibitors enhanced tear production and improved DED in rheumatoid arthritis^11^. The limited data in the literature hamper the recognition of these biologic agents’ real effects, specifically in DED associated with rheumatoid arthritis.

Therefore, this investigation aimed to evaluate the ocular surface parameters of rheumatoid arthritis patients and the impact of long-term TNF-inhibitors therapy on the ocular surface feature of rheumatoid arthritis patients, especially the evaluation of conjunctival goblet cell as a biomarker.

## Methods

### Subjects

This study was performed at the Rheumatology Division and the Department of Ophthalmology of the Hospital das Clinicas da Faculdade de Medicina, Universidade de Sao Paulo, Sao Paulo, Brazil. From July 2007 to October 2014, 16 consecutive rheumatoid arthritis patients, according to American College of Rheumatology/European League Against Rheumatism (ACR/EULAR) criteria^12^, aged ≥ 18 years and eligible to receive TNFi therapy due to joint symptoms were selected^13^.

The exclusion criteria included: secondary Sjögren’s syndrome, according to 2002 American-European Consensus Group^14^, smoking, active ocular disease such as allergy, infections, and glaucoma; use of topical lubricants or anti-inflammatory drugs (corticosteroids and cyclosporine A) or glaucoma treatment; recent ocular surgery; use of contact lenses. All clinical procedures fulfilled the tenets of the Declaration of Helsinki. The Local Research Ethics Committee (Comissão de Ética para Análise de Projetos de Pesquisa—CAPPesq do HCFMUSP, CAAE 37166914.8.0000.0068) approved the study, and all subjects accepted to participate in the study and signed an informed consent form.

At baseline, they were evaluated by experienced ophthalmologists and compared to a control group of healthy volunteers with similar age and gender. Blood samples were collected to measure erythrocyte sedimentation rate (ESR) and C-reactive protein (CRP), which are systemic inflammatory marker tests used to monitor and detect inflammatory disorders in both groups. Concomitantly, rheumatoid arthritis patients were assessed by rheumatologists before initiating TNFi treatment.

Rheumatoid arthritis patients were then prospectively re-evaluated with the same ophthalmological and rheumatologic parameters at three months (3 M). An additional evaluation was performed at 12 months (12 M) for those patients who persisted on TNFi.

### Ocular surface disease index (OSDI) questionnaire

Application of a self-reported questionnaire for DED (the Ocular Surface Disease Index—OSDI), culturally adapted, and validated for Portuguese occurred to all subjects^15^. This survey estimated dry eye symptoms, their impact on daily life activities, and environmental triggers. The score (range 0–100) discriminated among normal (0–12), mild (13–22) to moderate (23–32) and, severe (≥ 33) dry eye^16,17^.

The participants answered the questionnaire and underwent the ocular surface examination (Schirmer test, slit-lamp biomicroscopy, tear break-up time, ocular surface vital staining with fluorescein and lissamine green) at the baseline, 3 M, and 12 M evaluations.

### Ophthalmological examination

The ocular surface evaluation took place at the baseline visit (BL) in all patients and controls and, after three months (3 M) and 12 months (12 M), following the introduction of TNFi therapy.

Schirmer I test: all patients underwent Schirmer’s test without anesthesia. The strips stayed at the temporal third of the inferior eyelid of both eyes for 5 min. According to the scale (0–35 mm), the wet extension of the strips gave the value of the Schirmer test. Results below 10 mm were considered as diagnostic of dry eye^18^.

A full ophthalmological examination evaluated the ocular surface by slit-lamp biomicroscopy recording characteristics of eyelids, cornea, conjunctiva, and tear film. Meibomian gland dysfunction (MGD) was considered positive when there was: (1) meibomian orifice plugging or eyelid margin foaminess or hyperemia/telangiectasia at eyelid inspection or (2) a reduced or absent meibum secretion, or viscous to tooth-paste like secretion on digital pressure over the eyelid margins^19^.

Fluorescein strips (fluorescein 1.0 mg/mL, Ophthalmos, São Paulo, Brazil), wetted with 0.9% sodium chloride, and applied to the inferior fornix, measured tear break-up time (TBUT). Afterward, the patient blinked and stayed with eyes open: the chronometer or stopwatch (timepiece to measure the amount of time that elapses between the manual activation and deactivation) was activated, and the registration of the first dark spot was the TBUT, and the final TBUT was the mean of three measurements. Values below 10 s were considered as diagnostic of dry eye^18^.

**Figure 1:**
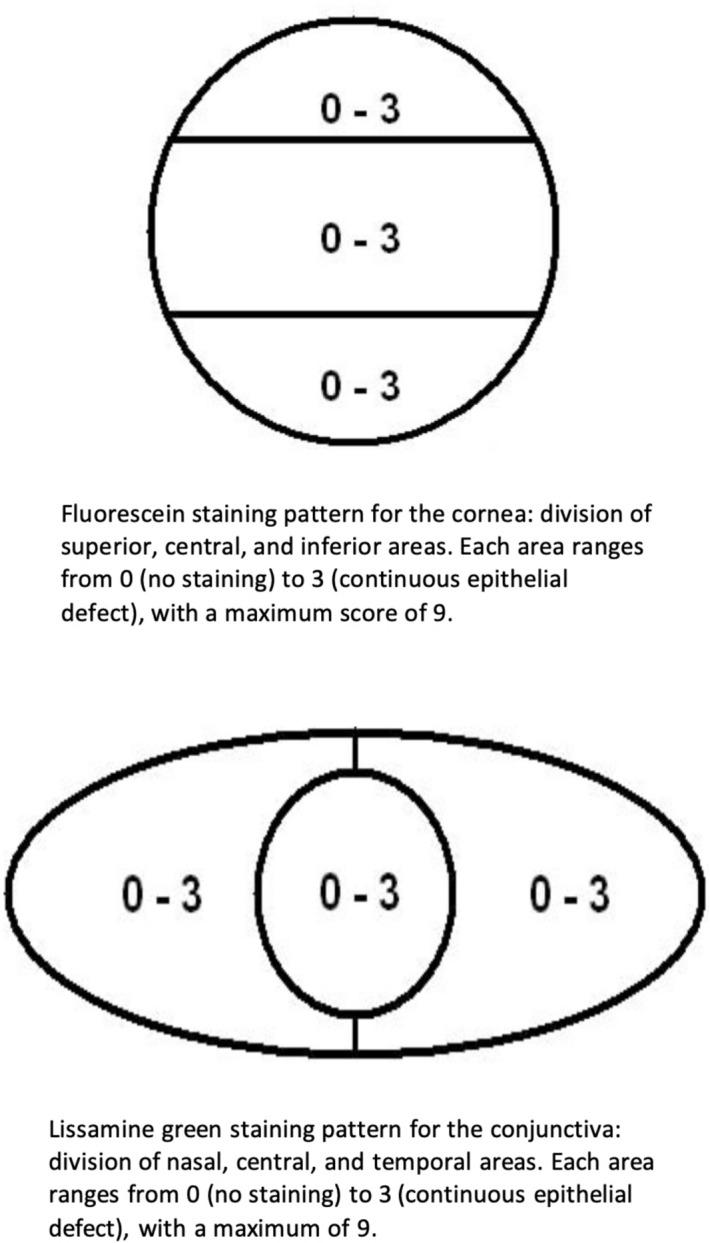
Diagram of ocular surface staining: divisions of the cornea (above) and conjunctiva (below) and respective scales.

To assess the ocular surface’s epithelial integrity and cell vitality, vital staining of the cornea and conjunctiva was performed using fluorescein, and lissamine green impregnated strips, respectively. Initially, fluorescein (strips containing fluorescein 1.0 mg/mL, Ophthalmos, São Paulo, Brazil) was applied, the corneal staining pattern was graded for the superior, central and inferior areas, in a score ranging from 0 (no staining) to 3 (continuous epithelial defect) and registered in a diagram. The total score was the sum of the three areas, with a maximum score of 9 (Fig. 1)^20^. Secondly, the lissamine green (strips containing lissamine green 1.5 mg/mL, Ophthalmos, São Paulo, Brazil) was applied using the same method, and the conjunctival staining was graded for the nasal, central and temporal areas, using the same criteria as the cornea, from 0 (no staining) to 3 (continuous epithelial defect), with a maximum score of 9 and registered in a diagram (Fig. 1)^20^. Values of a score above three were considered abnormal^21^.

From the measurements described above (symptoms and the value of the objective clinical signs), a dry eye severity grading scheme according to the Dry Eye Workshop (DEWS) classification method was applied^22^ by a masked observer. The status of severity ranged from 1 to 4.

Level 1 (mild): mild symptoms, no conjunctival or corneal staining, meibomian gland dysfunction (MGD) variably present and variable values of TBUT (second), and Schirmer scores (mm/5 min). The two latter parameters are less relevant for level 1 classification than mild symptoms or objective measurements. Level 2 (moderate)-moderate symptoms, a variable score of conjunctival and corneal staining, meibomian gland dysfunction variably present, BUT ≤ 10 s, Schirmer ≤ 10 mm/5 min. Level 3 (moderate/severe)-severe or frequent symptoms, moderate to marked conjunctival and corneal staining, meibomian gland dysfunction frequent, TBUT ≤ 5 s, Schirmer ≤ 5 mm/5 min. Level 4 (severe)-severe symptoms, severe conjunctival and corneal staining, meibomian gland dysfunction such as trichiasis, keratinization and symblepharon present, immediate TBUT, Schirmer ≤ 2 mm/5 min.

Participants presenting any level of DED severity were considered positive for DED.

All the ocular clinical measurements occurred in the same room, within the same day interval (from 1:00 PM to 4:00 PM) at the same conditions of illumination, temperature, and relative humidity^23,24^.

Patients presenting DED after the baseline evaluation received preservative-free lubricant eye drop four times a day and received instructions not to instill any eye drop on the day of their evaluation.

### Impression cytology of the ocular surface and goblet cell count

After all the ophthalmological examination, execution of impression cytology (IC) occurred at baseline, 3 M, and 12 M. Collection of impression cytology occurred on inferotemporal bulbar conjunctiva with cellulose acetate filters (Millipore GVWP, PVDF, 0.22 μm pore, Millipore Corp., Bedford, MA, USA) pressed for 10 s using the blunt end of a forceps under local anesthesia with 0.5% proparacaine hydrochloride. The specimens were fixed with absolute ethyl alcohol, dyed with periodic acid-Schiff (PAS)-hematoxylin stain, and were evaluated with light microscopy and graded into four stages according to Nelson grading system^25^. This method explored the epithelial cell morphology and nucleus to cytoplasmic ratio, goblet cell density, and goblet cell cytoplasm. Grade 0: epithelial cell size < 1.0 µm^2^, nucleus large; Nucleus: cytoplasm ratio 1:2, goblet cell > 500, and goblet cell cytoplasm PAS+ + +. Grade 1: epithelial cell size = 1.0 µm^2^, nucleus small; Nucleus: cytoplasm ratio 1:3, goblet cell 350–500, and goblet cell cytoplasm PAS+ +. Grade 2: epithelial cell size > 1.0 µm^2^, nucleus small; Nucleus: cytoplasm ratio 1:4–1:5, goblet cell 100–350, and goblet cell cytoplasm PAS++. Grade 3: epithelial cell size > 1.0 µm^2^, nucleus picnotic/absent; Nucleus: cytoplasm ratio 1:6, goblet cell < 100, and goblet cell cytoplasm PAS−. Grade 0 was considered normal, and grades 1, 2, and three, abnormal. Ten fields of each specimen were analyzed, and the most common classification was the final grade^26^.

For the goblet cell count (GCC), registration of density analysis utilized 13 high magnification fields for each sample^27^, since there is variability in the goblet cell density over the different areas of the material sample collected. At this magnification, the number of goblet cells from each counted area was multiplied by 1/0.036 (× 27.8) to result in an estimated goblet cell count per square millimeter^28^. The mean of the estimated goblet cells from the 13 areas was the goblet cell count. Record of all specimens succeeded under the same microscope (Nikon) at the same magnification (× 400), and the analysis was masked.

### Rheumatological examination

Baseline demographic and disease characteristics were recorded, including previous and current use of corticosteroids and synthetic and biological disease-modifying anti-rheumatic drugs (DMARDs), frequency of comorbidities, and extra-articular involvement. The evaluation of disease activity at baseline and during follow-up included clinical assessment by composite scores (Disease Activity Score in 28 joints using erythrocyte sedimentation rate—DAS28, clinical and simplified disease activity index—CDAI/SDAI), number of swollen and tender joints, patient’s global Health (PGA) and pain assessments and physician’s assessments of disease activity, measured by visual analogue scale (VAS) in cm and Health Assessment Questionnaire (HAQ) (Supplementary information)^29^. The inflammatory markers erythrocyte sedimentation rate (ESR) by the Westergren method and C-reactive protein (CRP) by nephelometry were also accessed longitudinally at RA patients before each scheduled visit. The TNFi used were intravenous infliximab (Remicade) 3 mg/kg at weeks 0, 2, and 6, and then every 6–8 weeks or subcutaneous etanercept 50 mg every week. For the prospective evaluations, patients were only included if they persisted in the same biologic treatment.

### Sample size

The study was exploratory with a convenience sample without a previous size estimation.

### Statistical analysis

Results are presented as median (interquartile range) and number (%) for continuous and categorical variables, respectively. Continuous variables were compared to the Mann–Whitney test to evaluate differences between rheumatoid arthritis and healthy controls, and by non-parametric Wilcoxon Signed Rank Test and Friedman Repeated Measures Analysis of Variance on Ranks test to evaluate rheumatoid arthritis patients in longitudinal analyses. Correlation analyses were performed using the Spearman correlation. For categorical variables, differences were assessed using Fisher’s exact test or chi-square test, as appropriate. In all statistical tests, the level of significance was set at 5% (*p* < 0.05). Licensed Stata/SE 14 was used as software.

## Results

### Baseline data

Rheumatoid arthritis patients and healthy controls had comparable age [49.5 (39.5–57) vs. 52 (47.5–55.5) years, *p* = 0.51], frequency of female sex (93.7% vs. 95.8%, *p* = 1.0) and frequency of Caucasians (87.5% vs. 70.8%, *p* = 0.27). Disease duration was 18 (9.5–24) years. Inflammatory markers CRP [10.1 (3.5–20.5) vs. 3.3 (3.3–3.3), *p* = 0.028] but not ESR [22 (7.5–38.5) vs. 16 (10.3–20), *p* = 0.25] was higher in rheumatoid arthritis patients when compared to controls. Rheumatoid factor (RF) was positive in 12 (75%) patients. Concomitant therapy at study entry were: 14 (85.7%) prednisone [dose 10 (7.5–10) mg/day], 13 (81.3%) methotrexate [dose 25 (20–25) mg/week], 8 (50%) leflunomide (dose 20 mg/day), 10 (62.5%) hydroxychloroquine (dose 400 mg/day) and 1 (6.3%) sulfasalazine (dose 3 g/day) mostly (n = 10; 62.5%) in combination of at least two conventional synthetic medicines. At study entry, 13 (81.2%) patients received infliximab, and 3 (18.8%) patients received etanercept. Table 1 illustrates the comparison of ocular parameters between rheumatoid arthritis patients and healthy controls at baseline. Symptoms score (OSDI) was higher (*p* = 0.007) in rheumatoid arthritis patients, who also presented a significantly lower median Schirmer score (*p* = 0.005) compared to controls (Table 1). The TBUT and ocular surface scores (fluorescein and lissamine green scores) were similar in both groups (*p* > 0.05). Meibomian gland dysfunction was more frequent in patients than in controls (*p* = 0.007). Rheumatoid arthritis patients also presented a higher frequency of abnormal scores of impression cytology (*p* < 0.001), higher impression cytology score itself (*p* < 0.001), and lower goblet cell count (*p* = 0.004). Dry eye parameters revealed a higher overall frequency of DED (*p* < 0.001), mostly mild DED when compared to controls (*p* < 0.001) (Table 1). The presence of meibomian gland dysfunction (MGD) was the only ocular parameter associated with most disease activity parameters at baseline (Table 2).

**Table 1:** Baseline ocular surface parameters of Rheumatoid Arthritis (RA) patients compared to healthy controls.

	RA patients (n = 16)	Control (n = 24)	*p*
OSDI (score)	22.9 (11.4–41.7)	1.0 (0–8.3)	**0.007**
OSDI abnomal, n (%)	11 (68.8)	4 (16.7)	**0.002**
Schirmer (mm/5 min)	14.5 (10–20)	27 (17–33)	**0.005**
Schirmer < 10 mm, n (%)	6 (37.5)	2 (8.3)	**0.042**
TBUT (seconds)	9 (6–12)	8 (6.5–10)	0.70
TBUT < 10 s, n (%)	11 (68.8)	15 (62.5)	0.68
Fluorescein (score)	0 (0–0)	0 (0–0)	0.99
Lissamine green (score)	0 (0–0)	0 (0–0)	0.65
MGD, n (%)	8 (50)	2 (8.3)	**0.007**
IC (score)	1 (0.5–2)	0 (0–0)	**< 0.001**
IC abnomal, n (%)	12 (75)	2 (8.3)	** 0.001**
GCC (cells/mm^2^)	325 (274–707)	742 (562–863)	**0.004**
Dry eye, n (%)	13 (81.3)	1 (4.2)	**< 0.001**
**Severity, n**			
Absent	3 (18.7)	23 (95.8)	
Mild	12 (75)	1 (4.2)	**< 0.001**
Moderate	1 (6.3)	0	

Data are expressed as medians (interquartile range) or absolute numbers (percentage). *RA* rheumatoid arthritis, *OSDI* Ocular Surface Disease Index (range 0–100), *mm* millimeters; Schirmer’s test range 0–35 mm, *TBUT* tear break-up time; Fluorescein and Lissamine green scores range 0–9, *MGD* meibomian gland dysfunction, *IC* impression cytology (range 0–3), *GCC* goblet cell count; Bold values: statistical significance = *p* < 0.05.

**Table 2:** Baseline disease activity parameters of RA patients, according to the presence of Meibomian Gland Dysfunction (MGD) (n = 16).

	Patients with MGD (n = 8)	Patients without MGD (n = 8)	*p*
DAS28 (score)	5.7 (5.6–6.8)	5.1 (3.4–6)	**0.027**
CDAI	38 (33.2–46.4)	27.7 (17.6–29.3)	**0.005**
SDAI	38.8 (34.3–49.9)	28.5 (17.7–31.6)	**0.007**
Tender joints	15.5 (10–17.5)	7.5 (5–10.5)	**0.031**
Swollen joints	9.5 (7–13.5)	8 (5.5–10)	0.23
Patient’s global health assessment (cm)	7.3 (5.6–8.7)	4.5 (2.6–5.4)	**0.004**
Pain assessment (cm)	6.5 (5–7.9)	3.6 (3.1–5)	**0.031**
Physician’s global assessment (cm)	7.3 (5.8–8.8)	5 (4.1–6.2)	**0.004**
HAQ-DI	1.9 (1.4–2.4)	0.4 (0.3–1.1)	**0.013**
ESR (mm/1st h)	22 (13–33)	21.5 (7–45.5)	0.66
CRP (mg/L)	10.1 (6.3–18.4)	8.3 (2.2–21.6)	0.65

Data are exposed as median (interquartile range), *DAS28* Disease Activity Score (range 0.49–9.07), *CDAI* clinical disease activity index (range 0–76), *SDAI* simplified disease activity index (range 0–86); Patient’s global health and pain assessments and Physician’s global assessments are evaluated using visual analoque scales (VAS) and ranges from 0 to 10 cm, *HAQ* Health Assessment Questionnaire (range 0–3), *ESR* erythrocyte sedimentation rate, *CRP* C-reactive protein; Bold values: statistical significance = *p* < 0.05.

### Prospective data

Table 3 shows the prospective evaluation of rheumatoid arthritis patients’ ocular and disease parameters at baseline and three months (3 M) of TNFi. All the 16 rheumatoid arthritis patients were already using the same initial TNFi. There was a statistically significant improvement in Schirmer’s test (*p* = 0.001), impression cytology score (*p* = 0.031), and goblet cell count (cells/mm^2^), reaching normal levels (*p* < 0.001). Other ocular parameters did not change (*p* > 0.05) during this period, but most of the articular disease activity improved (*p* < 0.05). The frequency of meibomian gland function did not change significantly over time, without association with clinical response.

**Table 3:** Prospective analysis of Rheumatoid Arthritis (RA) patients before and after 3 months (3 M) of TNFi therapy (n = 16).

	Baseline	3 M	*p*
**Ocular parameters**			
OSDI (score)	22.9 (11.4–41.7)	13.9 (5–19.4)	0.12
Schirmer (mm/5 min)	13 (7.5–17.5)	23.5 (16–35)	**0.001**
TBUT (s)	9 (6–12)	8.5 (6–10)	0.38
Fluorescein (score)	0 (0–0)	0 (0–0)	0.81
Lissamine green (score)	0 (0–0)	0 (0–0)	1
MGD (%)	8 (50)	8 (50)	1
IC (score)	1 (0.5–2)	1 (0–1)	**0.031**
GCC (cells/mm^2^)	325 (274–707)	931 (656–1,244)	** < 0.001**
**Disease activity parameters**			
DAS28 (score)	5.6 (5–6.1)	3.8 (2.9–4.9)	** < 0.001**
CDAI	31.1 (26.4–38)	16.3 (11.8–28.2)	**0.002**
SDAI	32.7 (28.2–38.8)	16.8 (12–28.4)	**0.002**
Tender joints	10 (6.5–16.5)	4.5 (1.5–9.5)	**0.007**
Swollen joints	8 (6.5–12)	5 (3–8)	**0.004**
Patient’s global health assessment (cm)	5.4 (4.5–7.3)	4.8 (3–6.5)	0.20
Pain assessment (cm)	5 (3.6–7.3)	3 (1.8–5.5)	0.05
Physician’s global assessment (cm)	5.9 (5.1–7.5)	3.2 (2.3–5.5)	**0.002**
HAQ-DI	1.4 (0.4–1.9)	1.1 (0.7–1.7)	0.40
ESR (mm/1st h)	22 (7.5–38.5)	12 (6–30.5)	0.11
CRP (mg/L)	10.1 (3.5–20.5)	2.6 (1.7–15.6)	0.32

Data are expressed as medians (interquartile range) or absolute numbers (percentage). 3 M: 3 months; *RA* rheumatoid arthritis, *OSDI* Ocular Surface Disease Index (range 0–100); mm = millimeters; Schirmer’s test range 0–35 mm; *TBUT* tear break-up time; Fluorescein and Lissamine green scores range 0–9, *MGD* meibomian gland dysfunction; *IC* impression cytology (range 0–3), *GCC* goblet cell count, *DAS28* Disease Activity Score (range 0.49–9.07), *CDAI* clinical disease activity index (range 0–76), *SDAI* simplified disease activity index (range 0–86); Patient’s global health and pain assessments and Physician’s global assessments are evaluated para visual analoque scales (VAS) and ranges from 0 to 10 cm, *HAQ* Health Assessment Questionnaire (range 0–3), *ESR* erythrocyte sedimentation rate, *CRP* C-reactive protein; Bold values: statistical significance = *p* < 0.05.

**Figure 2:**
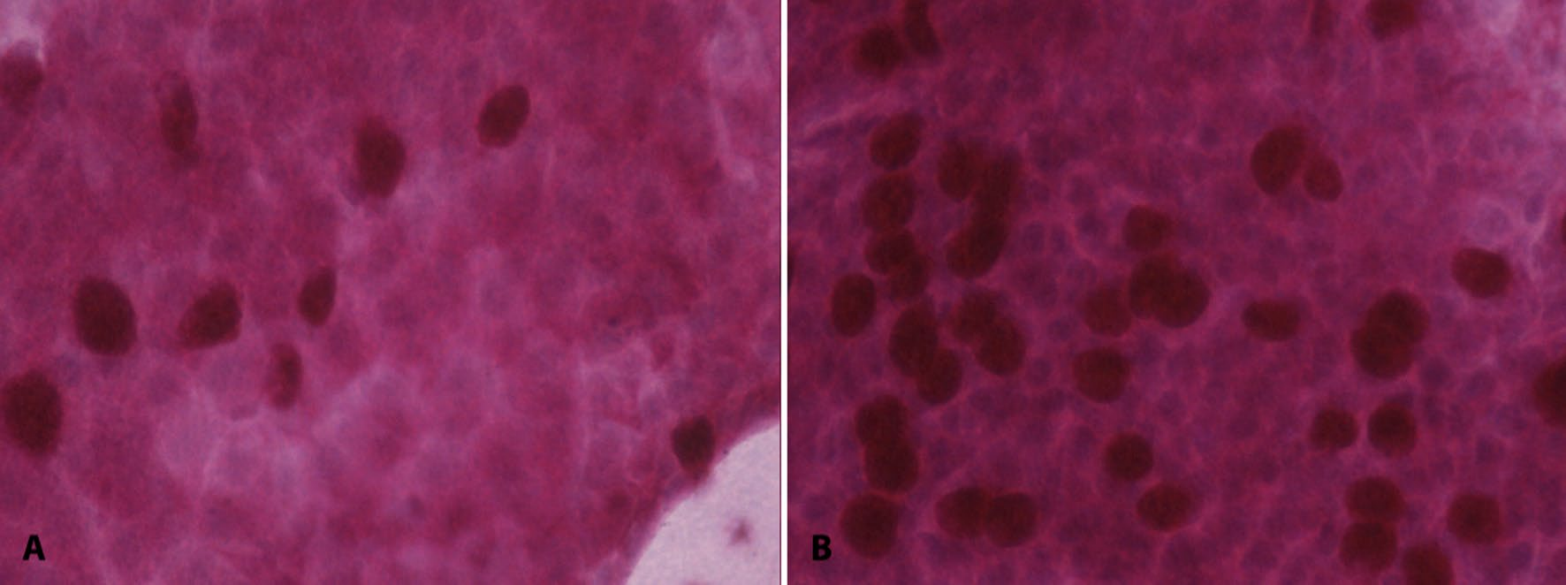
Conjunctiva Impression Cytology (IC) stained with periodic acid-Schiff (PAS)-hematoxylin stain of Rheumatoid Arthritis patients. (**A**) Baseline IC: grade 2 (100–350 cells/mm^2^—abnormal) according to Nelson’s classification IC. (**B**) 12 months of treatment with TNF-inhibitors: grade 0 (> 500 cells/mm^2^—normal), according to Nelson’s classification IC.

**Figure 3:**
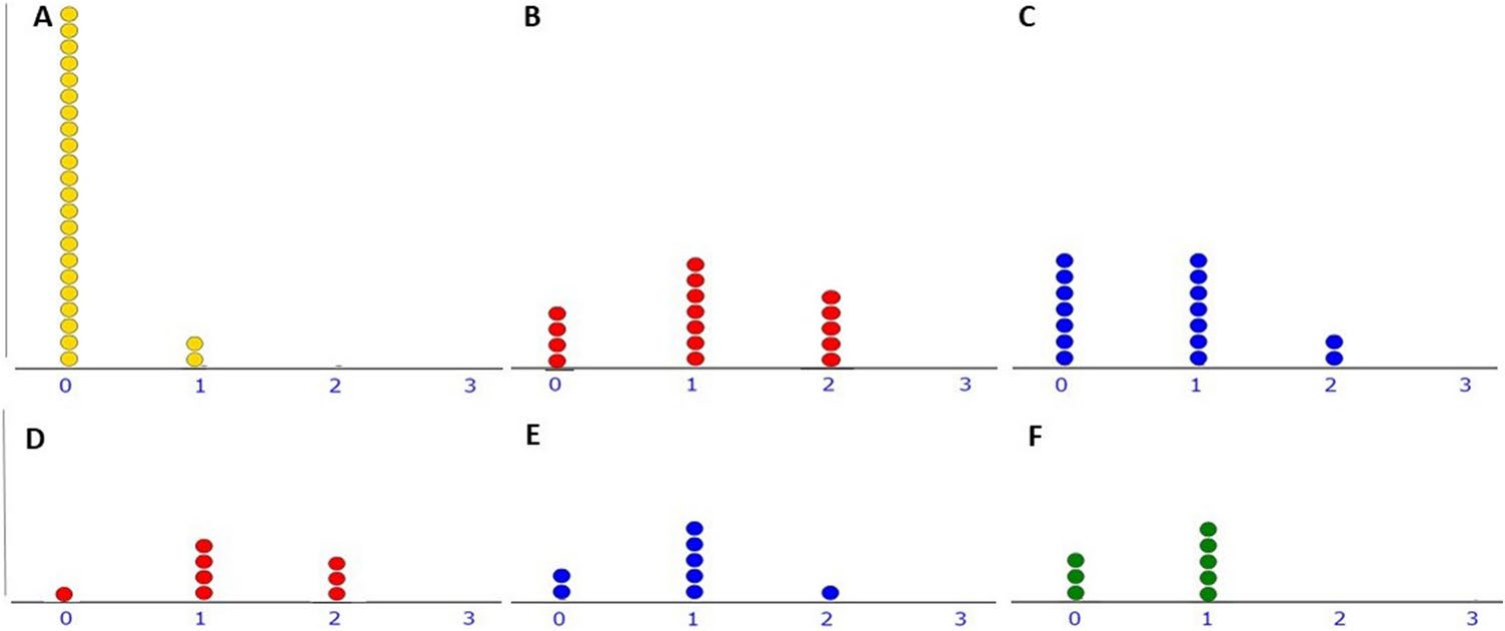
Dot plot of the number of patients (Y-axis) for each possible value of the impression cytology (IC) score (range 0–3: X-axis). (**A**) IC of healthy controls, (**B**) IC of 16 Rheumatoid Arthritis (RA) patients at baseline, and (**C**) IC of RA patients after 3 months of TNFi therapy. RA patients have higher scores than healthy controls at baseline (A vs. B; *p* < 0.001) and show a slight improvement after treatment (B vs. C, *p* = 0.031). From D to F: prospective IC scores of 8 RA patients who persisted on the same TNFi for 12 months. (**D**) IC at baseline; (**E**) IC at 3 months, and (**F**) IC at 12 months. A trend of improvement in the score is observed (*p* = 0.055).

After 12 months (12 M), only 8 patients (50%) persisted in the same TNFi initially prescribed and were re-evaluated: 7 on infliximab and 1 in etanercept. Table 4 shows the longitudinal evaluation of the ocular and disease parameters after 3 M and 12 M treatment of these patients. Goblet cell count (cells/mm^2^) was the only parameter with a progressive improvement reaching normal levels at 3 M (*p* < 0.001), but with an additional increase at 12 M (Fig. 2, 3). Other ocular parameters did not show statistically significant improvements during the study. Infections, peripheral ulcerative keratitis, and episcleritis did not occur during the period of follow-up. As expected, a significant improvement occurred in most of articular disease activity parameters (Table 4).

**Table 4:** Prospective analysis of Rheumatoid Arthritis (RA) patients before and after 3 (3 M) and 12 months (12 M) of TNFi therapy (n = 8).

	Baseline	3 M	12 M	*p*
**Ocular parameters**				
OSDI (score)	11.4 (0–31.3)	10.4 (1.3–14.2)	5 (0–16.8)	0.44
Schirmer (mm/5 min)	13 (10–25)	23 (13–30)	22.5 (16–26)	0.14
TBUT (seconds)	8 (6–12)	9 (5–11)	6.5 (5–8.5)	0.45
Fluorescein (score)	0 (0–0)	0 (0–0.5)	0 (0–0.5)	0.97
Lissamine green (score)	0 (0–0)	0 (0–0.5)	0 (0–1)	0.97
IC (score)	1 (1–2)	1 (0.5–1)	1 (0–1)	0.055
GCC (cells/mm^2^)	393 (275–827)	872 (502–1,185)	1,079 (867–1,244)	**0.047**
**Disease activity parameters**				
DAS28 (score)	5.1 (3.4–5.9)	2.9 (1.8–3.7)	2.3 (1.8–3.8)	**0.03**^+^
CDAI	29.3 (17.6–35.3)	11.8 (7.9–16.3)	8 (6.3–13.8)	< 0.001*^+^
SDAI	30.3 (17.7–36.4)	12 (8.6–16.5)	8.4 (6.3–14.3)	< 0.001*^+^
Tender joints	9 (5–13)	1.5 (1–3.5)	1.5 (1–5.5)	**0.047**
Swollen joints	8 (7–11)	3.5 (2–6)	0.5 (0–3.5)	< 0.001*^+^
Patient’s global health assessment (cm)	4.5 (2.6–6.8)	3 (1.9–3.9)	3.6 (1.5–5.8)	0.65
Pain assessment (cm)	4.3 (3.4–7.1)	1.8 (1.1–2.4)	4 (2.4–7.1)	0.002*^+^
Physician’s global assessment (cm)	5.4 (4.1–6.2)	3 (2.3–3.3)	1.6 (1.1–3.1)	0.002*^+^
HAQ-DI	1 (0.3–1.6)	0.8 (0.5–1.3)	0.7 (0.4–1.3)	0.17
ESR (mm/1st h)	9 (7–22)	6 (3.5–20)	5 (3–12.5)	0.15
CRP (mg/L)	3.5 (2–11.7)	1.9 (0.6–3.8)	2.7 (0.8–6.9)	0.47

Data are expressed as medians (interquartile range) or absolute numbers (percentage). 3 M: 3 months; 12 M: 12 months; *RA* rheumatoid arthritis, *OSDI* Ocular Surface Disease Index (range 0–100), *mm* millimeters; Schirmer’s test range 0–35 mm, *TBUT* tear break-up time; Fluorescein and Lissamine green scores range 0–9, *MGD* meibomian gland dysfunction, *IC* impression cytology (range 0–3), *GCC* goblet cell count, *DAS28* Disease Activity Score (range 0.49–9.07), *CDAI* clinical disease activity index (range 0–76), *SDAI* simplified disease activity index (range 0–86); Patient’s global health and pain assessments and Physician’s global assessments are evaluated para visual analoque scales (VAS) and ranges from 0 to 10 cm, *HAQ* Health Assessment Questionnaire (range 0–3), *ESR* erythrocyte sedimentation rate, *CRP* C-reactive protein; Bold values: statistical significance = *p* < 0.05; *p < 0.05 between BL versus 3 M; ^+^*p* < 0.05 between BL versus 12 M.

There was no association among disease activity and dry eye parameters, neither between signs and symptoms of DED.

## Discussion

This study evaluated rheumatoid arthritis patients with mild severity dry eye disease and demonstrated that aqueous (Schirmer), lipid (meibomian gland dysfunction), and mucin (goblet cells count) components of dry eye are present in such population. Treatment with TNF inhibitors improved conjunctival goblet cells count since the third month of therapy, up to 12 months. For the first time, the evaporative component of dry eye evaluated by the presence of meibomian gland dysfunction was associated with high rheumatoid arthritis disease activity.

The present study’s paramount advantage was the use of the most recent validated dry eye clinical criteria and severity grading system, including signs and symptoms^30,31^. This approach increases sensitivity for dry eye diagnosis compared to the Schirmer test or conjunctival staining (rose bengal or lissamine green) employed in the Sjögren’s syndrome (SS) classification^14,21^. This severity grade system results in better diagnostic accuracy, with enhanced early disease detection and distinction of severity grades^22,31^. Such a method also allows for a more precise therapeutic strategy for each group of patients^32^. Moreover, there was a rigorous control of temperature and relative humidity conditions, which is crucial for dry eye evaluation once these external environmental factors influence dry eye variability^23,24^.

More than three-quarters of the patients enrolled in this study presented DED, most of them with mild severity. Aluru et al. found a comparable prevalence of 86% of DED in rheumatoid arthritis patients (most of them with moderate to severe grade). However, they selected DED patients from a tertiary eye center while we evaluated asymptomatic rheumatoid arthritis patients with previously undiagnosed DED. They classified their patients using only objective signs of DED based on modified DEWS criteria for severity grading, whereas our approach also included the evaluation of symptoms^33^.

This presentation with predominant mild DED in previously asymptomatic rheumatoid arthritis patients completes the spectrum grade with a low to intermediate result, differently from what is commonly studied: subjects have been explicitly chosen to represent a DED cohort. Most of the studies use subgroups of patients/individuals to characterize the two extremes of DED severity measure distribution (Sjögren’s syndrome-dry eye and healthy controls)^34^. Nevertheless, studying only severe cases can underestimate the impact of mild forms of DED in quality of life, the opportunity to understand physiopathology, and to treat early disease, avoiding severe complications.

For the first time, we demonstrated the evaporative component disturbance in rheumatoid arthritis patients, with the presence of meibomian gland dysfunction (associated with alterations in the lipid layer of tear film) as well as decreased goblet cells count (associated with the mucin layer of the tear film), in addition to the traditional aqueous tear deficiency related to keratoconjunctivitis sicca. The combined aqueous deficient and evaporative dry eye is in line with previous reports in primary SS and current guidelines for DED evaluation^18,32^.

In fact, meibomian gland dysfunction is the most common factor contributing to the evaporative dry eye that, in turn, is more frequent than the aqueous-deficient dry eye^23^. The gland dysfunction (either by a blockage, drop-out, or inflammation) participates in the vicious circle of dry eye disease’s pathophysiology: leaving to a deficit of tear film lipid layer and increased tear evaporation and inflammation which is part of the core mechanism of dry eye^35^. The association of meibomian gland dysfunction and rheumatoid arthritis disease activity parameters shows that the evaporative component of dry eye is not a mere finding but is part of the rheumatic disease’s pathophysiologic process. It is in line with Fujita et al.’s previous findings that observed a relationship between dry eye and disease activity in rheumatoid arthritis patients. However, they used an alternative classification of DED as well as the unconventional Lansbury index to evaluate disease activity in rheumatoid arthritis^3^. In contrast, Aluru et al. did not find an association between the severity of DED with the severity of the rheumatoid arthritis^33^.

The mucin-secreted component forming the tear film’s glycocalyx is not routinely investigated in dry eye patients, even presenting a vital function for the tear composition. Recently, the ODISSEY European Consensus Group included the goblet cell count as an objective marker for the DED diagnosis^31^, and the Tear Film and Ocular Surface Dry Eye Workshop II (TFOS DEWS II) included the impression cytology as a reference to ocular surface damage^36^. Freshly, goblet cells comply with requirements as a critical biomarker in ocular matrices^37^. Impression cytology collects superficial conjunctival layers of the ocular surface, is easily repeatable and collectible, and minimally invasive^26,28,36^. The goblet cell counts analyzed in impression cytology fulfilled the BEST (Biomarkers, Endpoints, and other Tools) Resource once it indicated normal or pathogenic process as well as response to treatment^37^. Furthermore, conjunctival goblet cells (by MUC5AC, a secreted gel-forming soluble mucin) promote lubrication, wetting, and form a mucosal barrier preventing infections, as having a role in ocular surface homeostasis^38^. The functional abnormality or the decrease in the number of goblet cells harm vision and ocular health^39^.

However, we did not demonstrate the association of goblet cell loss and vital dye staining. Such association was notably reported in primary SS with a more severe aqueous-deficient dry eye, but not for other causes of dry eye^40^. In line with this finding, our data suggest that a mild loss of goblet cells can represent a decrease of secreted-soluble mucin (MUC5AC), without compromising the production of transmembrane mucins from other conjunctival epithelium cells. Consequently, a preserved glycocalyx barrier would result in negative staining. Lissamine green, as a vital dye, solely stains epithelial cells with damaged membranes independently of the presence of mucin^36,41^. Furthermore, the ocular surface vital staining reduction with dry eye treatment is dependent on the severity of the baseline vital staining score^42^. Likewise, the same lack of correlation with goblet cell loss and TBUT was observed, concurring with published studies^9^. In fact, there is not a continuous linear relationship between these parameters: the TBUT depends on MUC5AC produced by goblet cells as well as other mucin produced by other conjunctival cells^9^. The cohort of rheumatoid arthritis patients composed basically of the female gender over 40 years old, contributing factors for dry eye disease, presented mild dry eye. The autoimmune disease and inflammation have a central role in this dry eye group^8,9^. The lissamine green and fluorescein staining representing damages of the ocular surface did not occur throughout the follow-up, probably due to the ocular surface cells’ inflammation control and homeostasis^41^.

Of note, rheumatoid arthritis patients had elevated C-reactive protein (CRP) and lower goblet cell density compared to healthy subjects. The altered conjunctival cytology and reduced goblet cell counts reflect this inflammation at the ocular surface since TNF-α and IFN-ɣ induce apoptosis of goblet cells in experimental animals and dry eye patients^43–45^. However, according to previous reports, erythrocyte sedimentation rate (ESR) of rheumatoid arthritis patients was not even elevated, and there was not any association among laboratory parameters and abnormal cytology, probably due to the use of concomitant prednisone and high dose methotrexate and other conventional synthetic disease-modifying drugs, especially in combination, at baseline^46,47^.

Moreover, present data suggest that TNFi treatment improved inflammatory disease activity concomitantly to conjunctival cytology, especially the goblet cell density, implying a common underlying inflammatory mechanism for ocular and articular activity. Of note, TNF-α was one of the central inflammatory mediators in rheumatoid arthritis joints and tear film^48^. Therefore, systemic therapeutic strategies of rheumatoid arthritis may be beneficial for ocular involvement associated with this disease when the mechanism of these conditions overlap^48^. The observed increase in conjunctival goblet cell count after TNF-inhibitors therapy indicates an improvement of ocular surface inflammation and could be considered a biomarker for treatment intervention^37^. In contrast, artificial tears, used as a symptomatic treatment in DED, do not affect goblet cell density^49^. Furthermore, Moore et al. evaluated dry eye patients using artificial tears after an acute ocular response to low-humidity environments. This treatment did not show an improvement in corneal or conjunctival staining neither on irritation symptoms but the use of topical corticosteroids did^50^.

The lack of association of symptoms and clinical signs measured by objective ocular parameters and the absence of improvement in this questionnaire during the period follow-up are in accordance to previous studies demonstrating that up to 60% of patients with altered tear break-up time, Schirmer test, corneal and conjunctival staining, meibomian gland dysfunction, and OSDI might not have DED symptoms^23,51^. Symptoms were considered to be characteristic of dry eye disease, but recent studies show that less than 60% of patients are symptomatic, and the use of symptoms alone for diagnosis of dry eye disease as well as isolated inclusion criteria will miss a significant percentage of dry eye patients^23^. The chronic surface damage of DED can cause impairment of corneal sensitivity, which can hide discomfort^22^. In patients with mild DED, as the present population, the lack of association with symptoms can be even more pronounced^23^.

This study’s main limitation is the small sample size since patients had a mild dry eye disease. The evaluation of some aspects was prone to underestimation, like TBUT, fluorescein, and lissamine green scores, which were comparable to the control group at baseline and did not improve during the follow-up. The study was also underpowered to refute definitively prospective variations of OSDI, meibomian gland dysfunction (MGD), and long-term evaluation of Schirmer and IC scores.

In conclusion, we identified that DED is frequent in rheumatoid arthritis patients, and it is multifactorial, comprising aqueous, lipid, and mucin components. The meibomian gland dysfunction is associated with higher disease activity parameters, and TNFi therapy improves ocular surface health by inducing recovery of goblet cells, which can be a conjunctival biomarker of the pathological process and response to therapy in DED. Additionally, systemic TNFi can be a treatment option for ocular surface inflammation in systemic inflamed rheumatoid arthritis patients.

## Supplementary information

Supplementary file1

## Data Availability

The datasets generated and analyzed during the current study are available from the corresponding author on reasonable request.
