# Supplementary material for: Dry eye in rheumatoid arthritis patients under TNF-inhibitors: conjunctival goblet cell as an early ocular biomarker
Source: Sci Rep. 2020 Aug 20;10:14054. doi: 10.1038/s41598-020-70944-9 (PMC7441175; doi:10.1038/s41598-020-70944-9)
Supplement: Supplementary file 1 — Supplementary file1 [file 41598_2020_70944_MOESM1_ESM.pdf]

# SUPPLEMENTARY INFORMATION

**DRY EYE IN RHEUMATOID ARTHRITIS PATIENTS UNDER TNF-INHIBITORS: CONJUNCTIVAL GOBLET CELL AS AN EARLY OCULAR BIOMARKER.**


Fany Solange Usuba [1], Ana Cristina de Medeiros-Ribeiro [2], Priscila Novaes [1], Nadia Emi Aikawa [2], Karina Bonfiglioli [2], Ruth Miyuki Santo [1], Eloisa Bonfá [2], Milton Ruiz Alves [1]

(1) Department of Ophthalmology, Hospital das Clinicas HCFMUSP, Faculdade de Medicina, Universidade de São Paulo, são Paulo, BR.

(2) Rheumatology Division, Hospital das Clinicas HCFMUSP, Faculdade de Medicina, Universidade de São Paulo, são Paulo, BR.


### Rheumatoid Arthritis diasease activity parameters

| | |
|---|---|
| Disease activity score (28 joints) - counts and natural logarithmic DAS28 (score with 4 variables)<br>(Varies from 0.49 to 9.07) | Calculation formula (requires a calculator), with square root (sqrt) of joint transformation (ln) of erytrocyte sedimentation rate (ESR).<br>= 0.56 * sqrt (tender joints – 28 possible described bellow)<br>+ 0.28 * sqrt (swollen joints – 28 possible described bellow)<br>+ 0.70 * ln (ESR – 2 to 100 mm/h)<br>+ 0.014× PGHA (patient's global health assessment – 0 to 100 milimeters) |
| Clinical Disease Activity Index -<br>CDAI (from 0 to 76) | Simple sum: swollen joints + tender joints (both from 28 possible) + patient´s assessement + physician´s assessement of disease activity (visual analogue scale in cm). |
| Simplified disease activity Index -<br>SDAI (from 0.1 to 86) | CDAI plus C-reactive protein (CRP) (0.1-10 mg/dl) |
| Tender joints (from 0 to 28) | Sum of tender joints among 28 possible: 2 wrists, 10 metacarpophalangeal joints, 8 proximal interphalangeal joints of hands, 2 thumb interphalangeal,joints, 2 elbows, 2 shoulders, and 2 knees |
| Swollen joints (from 0 to 28) | Sum of swollen joints among the same 28 possible joints |
| Patient´s global health assessment (PGHA) | Visual analogue scale from 0-10 (cm) (0-best; 10-worst) (for DAS28, register in mm – 0 to 100mm) |
| Pain assessement | Visual analogue scale from 0-10 (cm) (0-best; 10-worst) |
| Physician´s assessment of disease activity | Visual analogue scale from 0-10 (cm) (0-best; 10-worst) |

mm = milimetre; cm = centimetre. For all, the higher scores reflect worst disease activity


Ref.: http://www.das-score.nl/

Aletaha D, Smolen JS. The Simplified Disease Activity Index (SDAI) and Clinical Disease Activity Index (CDAI) to monitor patients in standard clinical care. *Best Pract Res Clin Rheumatol*. 21, 663–675 (2007). doi:10.1016/j.berh.2007.02.004

Visual Analogue Scales (can be registered in cm or mm) - The higher scores reflect worst disease activity

**Pain assessement**

How much pain have you had because of your illness IN THE PAST WEEK: place a vertical (|) mark on the line to indicate the severity of the pain

No Pain Severe Pain

|________________________________________________________________________ |

0 10

**Patient´s global health assessment (PGHA)**

Considering all the ways that your arthritis affects you, rate how you are doing on the following scale by placing a vertical mark on the line.

Very Well Very Poor

|________________________________________________________________________|

0 10

**Physician´s assessment of disease activity**

Considering all the ways that arthritis affects the patient, how well is he/she doing? Mark on the following scale by placing a vertical mark on the line.

Very Well Very Poor

|________________________________________________________________________|

0 10

Evaluation of disability – we used the Brazilian version of The STANFORD HEALTH ASSESSMENT QUESTIONNAIRE (HAQ-DI)

The English version of the questionnaire is bellow, with 8 domains. For each domain, register the highest score. The result is simple mean of the 8 domains.

| Please check the response which best describes your usual abilities OVER THE PAST WEEK: | | | | | |
|---|---|---|---|---|---|
| | Without ANY difficulty | With SOME difficulty | With MUCH difficulty | UNABLE to do | Register the highest score for each area (0-3) |
| | Score 0 | Score 1 | Score 2 | Score 3 | |
| **DRESSING & GROOMING** Are you able to: | | | | | |
| Dress yourself, including tying shoelaces and doing buttons? | | | | | |
| Shampoo your hair? | | | | | |
| **ARISING** Are you able to: | | | | | |
| Stand up from a straight chair? | | | | | |
| Get in and out of bed? | | | | | |
| **EATING** Are you able to: | | | | | |
| Lift a full cup or glass to your mouth? | | | | | |
| Open a new milk carton? | | | | | |
| Cut your meat? | | | | | |
| **WALKING** Are you able to: | | | | | |
| Walk outdoors on flat ground? | | | | | |
| Climb up five steps? | | | | | |
| **HYGIENE** Are you able to: | | | | | |
| Wash and dry your body? | | | | | |
| Take a tub bath? | | | | | |
| Get on and off the toilet? | | | | | |
| **REACH** Are you able to: | | | | | |

| | | | | | |
|---|---|---|---|---|---|
| Reach and get down a 5pound object (such as a bag of sugar) from just above your head? | | | | | |
| Bend down to pick up clothing from the floor?: | | | | | |
| **GRIP** Are you able to: | | | | | |
| Open car doors? | | | | | |
| Open jars which have been previously opened? | | | | | |
| Turn faucets on and off? | | | | | |
| **ACTIVITIES** Are you able to: | | | | | |
| Run errands and shop? | | | | | |
| Get in and out of a car? | | | | | |
| Do chores such as vacuuming or yardwork? | | | | | |
| | | | | **Final score** | |

Please check any AIDS OR DEVICES that you usually use for any of these activities:

| | | | |
|---|---|---|---|
| ( ) Cane | ( ) Devices used for dressing (button hook, zipper pul longhandled shoe horn, etc.) | ( ) Raised toilet seat | ( ) Long-handled appliances for reach |
| ( ) Walker | ( ) Built up or special utensils | ( ) Bathtub seat | ( ) Long-handled appliances in bathroom |
| ( ) Crutches | ( ) Special or built up chair | ( ) Jar opener (for jars previously opened) | ( ) Special or built up chair |
| ( ) Wheelchair | ( )Other (Specify: ) | ( ) Bathtub bar | ( ) Other (Specify: ) |

Please check any categories for which you usually need HELP FROM ANOTHER PERSON:

| | | | |
|---|---|---|---|
| ( ) Dressing and Grooming | ( ) Arising | ( ) Eating | ( ) Walking |
| ( ) Hygiene | ( )Reach | ( ) Gripping and opening things | ( ) Errands and chores |

Ferraz MB, Oliveira LM, Araujo PM, Atra E, Tugwell P. Crosscultural reliability of the physical ability dimension of the health assessment questionnaire. *J Rheumatol*. 17, 813–817 (1990).
